# Long-term impact of postconditioning on infarct size and left ventricular ejection fraction in patients with ST-elevation myocardial infarction

**DOI:** 10.1186/1471-2261-13-22

**Published:** 2013-03-25

**Authors:** Peder Sörensson, Lars Rydén, Nawsad Saleh, Per Tornvall, Håkan Arheden, John Pernow

**Affiliations:** 1 Karolinska Institutet, Department of Medicine, Unit of Cardiology, Karolinska University Hospital, Stockholm, Sweden; 2 Lund University, Department of Clinical Physiology, Skane University Hospital, Lund, Sweden; 3 Department of Cardiology, Karolinska University Hospital, Stockholm, 171 76, Sweden

**Keywords:** Myocardial infarction, Infarct size, Postconditioning, CMR

## Abstract

**Background:**

Ischemic postconditioning (PostC), reperfusion in brief cycles, is known to induce short-term reduction in infarct size in patients with ST elevation myocardial infarction (STEMI), especially among those with large myocardium at risk (MaR). The aim of the present study was to investigate the long-term effect of PostC on infarct size and left ventricular ejection fraction (LVEF).

**Methods:**

Sixty-eight patients with a first STEMI were randomised to primary percutaneous coronary intervention (PCI) (n = 35) or PCI followed by PostC (n = 33). MaR was determined as abnormally contracting segments on left ventricular angiogram. Cardiac magnetic resonance was performed at 3 and 12 months for the determination of infarct size and LVEF.

**Results:**

Overall there was no difference in infarct size expressed in percentage of MaR between patients randomised to the control (31%; 23, 41) and PostC (31%; 23, 43) groups at 12 months. Likewise there was no difference in LVEF between control (49%; 41, 55) and PostC (52%; 45, 55). In contrast, patients in the PostC group with MaR in the upper quartile had a significantly smaller infarct size (29%; 18, 38) than those in the control group (40%; 34, 48; p < 0.05) at 12 months. In these patients LVEF was higher in the PostC (47%; 43, 50) compared to the control group (38%; 34, 42; p < 0.01).

**Conclusions:**

In this long-term follow-up study PostC did not reduce infarct size in relation to MaR or improved LVEF in the overall study population. However, the present data suggest that PostC exerts long-term beneficial effects in patients with large MaR thereby extending previously published short-term observations.

**Trial registration:**

Karolinska Clinical Trial Registration (http://www.kctr.se). Unique identifier: CT20080014

## Background

Myocardial infarction (MI) remains a major health problem despite substantial improvements in detection and treatment [1]. Infarct size is a major determinant of subsequent mortality and morbidity [2,3]. Accordingly, therapeutic strategies aimed at limiting infarct size are of great prognostic importance in addition to current management strategies focused on early revascularisation with thrombolysis or primary percutaneous coronary intervention (PCI) stabilization [4]. Although opening of the infarct-related artery is beneficial it also initiates a series of harmful events including release of reactive free oxygen species and calcium overload that triggers the opening of the mitochondrial permeability transition pore (mPTP) [5]. This contributes to further myocyte necrosis and apoptosis, the so called reperfusion injury [6]. The reperfusion injury opens for therapeutic interventions aiming at myocardial protection with limitation of the final infarct size. Accumulating evidence suggests that postconditioning (PostC), repetitive brief cycles of ischemia and reperfusion during early reperfusion, reduces infarct size in experimental and clinical studies of patients with ST-elevation myocardial infarctions (STEMI) [7-11]. The main underlying mechanism seems to be inhibition of the mPTP through different pathways of kinases involving the reperfusion injury salvage kinase and the survivor activating factor enhancement pathway within the myocyte [12,13].

Previous studies employing PostC have mainly had short-term follow-up periods evaluating either plasma biomarkers of myocardial injury (creatine kinase or troponins) [9], infarct size [7,8] or left ventricular ejection fraction (LVEF) [14]. However, data concerning long-term (≥ 12 months) follow-up of patients treated with PostC are limited and no study has previously determined the effect of PostC on infarct size and LV function using cardiovascular magnetic resonance (CMR) imaging during a period of one year in patients with STEMI. The aim of the present study was therefore to investigate the long-term effect of PostC on infarct size in patients with STEMI in a randomised study. We have previously demonstrated that PostC reduced infarct size determined by CMR among patients with large myocardium at risk (MaR) [8]. The present study is a pre-specified follow-up on infarct size, LVEF, cardiac volumes and remodeling.

## Methods

### Study group

During April 2007 and March 2009 a total of 795 patients were referred to the coronary care unit at Karolinska University Hospital in Stockholm for a PCI due to STEMI of whom 89 were randomised and 76 completed the study protocol [8]. Patients were eligible for enrolment if they fulfilled the following inclusion criteria: men and women older than 18 years, chest pain >30 min and <6 h, ST elevation >0.1 mV (>0.2 mV in V1-V3) in two contiguous ECG leads or left bundle branch block, and a thrombolysis in myocardial infarction (TIMI) grade 0 flow in the infarct related artery. Exclusion criteria were previous myocardial infarction, previous coronary artery bypass surgery, cardiogenic shock, contraindication for CMR and persistent atrial fibrillation. A biplane (30º right anterior oblique, 60º left anterior oblique) left ventriculography was performed before revascularisation and MaR was estimated by measuring the circumferential extent of abnormally contracting segments [15]. Following this the patients were randomized to primary PCI only or PCI followed by PostC, in blocks of eight, following stratification for LAD and non-LAD occlusions. PostC was performed by reinflating the balloon at the same location to a pressure of 2–4 atm for 60 s starting 60 s after the initial reperfusion. This cycle was performed four times. The PCI intervention was completed by a coronary angiogram to study the final TIMI flow. Collateral flow to the infarct zone was assessed on the initial angiogram before PCI and graded on a scale of 0 to 3 [16]. All patients that underwent complete CMR protocol at three and 12 months were included in the final analysis. The study was performed according to the Declaration of Helsinki and good clinical practice [17]. Written informed consent was given by all patients. The study was approved by the local ethics committee at the Karolinska Institutet.

### CMR protocol

A standard CMR examination was performed after three and 12 months. These investigations were performed in the supine position with an 8-channel cardiac coil by means of a 1.5 T system (Signa Excite TwinSpeed, General Electric Healthcare, Waukesha, Wisconsin, USA) during vector-ECG monitoring. Gadolinium contrast (0.2 mmol/kg; Omniscan, GE Healthcare) was administered before positioning the patient in the scanner. The image protocol included scout images, localisation of the short axis and then covering the whole left ventricle (LV) with retrospectively gated cine steady-state free precession (SSFP) images. The following parameters were typically used; SSFP (echo time (TE) 1.58 ms, repetition time (TR) 3.61 ms, flip angle 60º, 25 phases, 8 mm slice, no gap, matrix, 226 × 226). Late gadolinium enhancement (LGE) images were acquired 15–20 min after contrast injection using an inversion recovery gradient echo sequence (TE 3.3 ms, TR 7.0 ms, inversion time 180–250 ms to null the myocardium, 8 mm slice, no gap, matrix 256 × 192) in the same slice orientation as cine SSFP images. Each slice was obtained during end-expiratory breath holding. Two-, three- and four-chamber views were also obtained to confirm the findings.

### Data analysis

All CMR images were analysed blinded and off-line using a freely available segmentation software (Segment V.1.8 R1405; http://segment.heiberg.se/) [18]. End-diastolic and end-systolic volumes were measured in the phase with the largest and smallest LV volumes respectively. LVEF, stroke volume and LV mass were calculated on cine SSFP sequences using manual delineation of the endocardial and epicardial borders. The papillary muscles were excluded from the myocardium. For correct LV volumes and mass estimations the basal slices were examined in different cine projections. LV mass was calculated by multiplying the myocardial volume by the density of myocardial tissue (1.05 g/ml). All volumes were indexed to body surface area. Infarct size was quantified on LGE images using an automated quantification method validated ex- and in vivo and in which partial volume effects were accounted for [19]. Manual adjustments were made when the computer algorithm was obviously wrong. Infarct size was than related to MaR for each patient. LGE images were also used for quantifying microvascular obstruction defined as hypoenhanced region within the infarct area. Results regarding infarct size in relation to MaR and LVEF on day 6–9 have already been published earlier while this report presents data from three and 12 months [8], LV remodeling was defined as an increase in end-systolic LV volume ≥15% from the value obtained one week after admission. Change in LV sphericity index was used for detection of cavity remodeling [20]. The major axis was manually measured in an end-diastolic four-chamber view starting at the mitral annulus and ending at the apical endocardial border. The radius was used for calculating the sphere volume. End-diastolic LV volume was divided by the sphere volume creating a sphericity index for every patient.

### Statistical analysis

All quantitative data are presented as median and inter quartile range. The Mann–Whitney U test was used to test for differences in infarct size and cardiac volumes and function between groups. Fisher’s exact test was used to test for differences between dichotomized variables. Linear regression was used for comparing infarct size in relation to MaR between groups and then the difference in slopes and intercepts between groups were tested (Infarct size = Intercept + Treatment (0/1) + MaR + Treatment (0/1)*MaR). Wilcoxon sign rank test with Bonferroni correction for multiple testing was used for longitudinal follow-up on CMR measurements. Bland-Altman plots were constructed for comparing intra- and interobserver variation and calculated as the SD of the difference between two calculations divided by the average of the two observers. Statistical analysis was performed using GraphPad Prism V 5.0 (GraphPad Software, San Diego, California, USA) and all tests were 2-tailed with a 5% significance level.

## Results

### Study group

**Figure 1:**
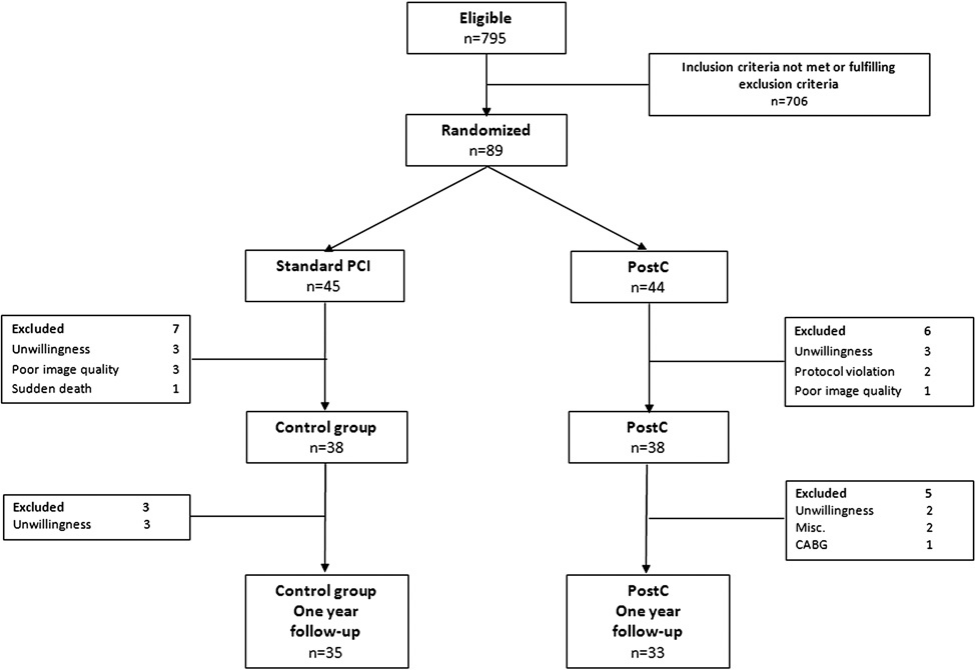
Flow chart.

Sixty-eight patients (35 in the control group and 33 in the PostC group) completed follow-up with CMR at three and 12 months. The main reason for loss to follow-up was unwillingness to complete the study protocol (Figure 1). No sudden cardiac death or myocardial infarction occurred in either group during follow-up. Five patients (4 controls) were hospitalized due to congestive heart failure (n = 3) or chest pain (n = 2) during the follow-up period. All patients were asymptomatic at the time of follow-up CMR and remained on the medication prescribed at the time of hospital discharge with the exception of clopidogrel, which was terminated after three months in case of bare metal stent implantation. As seen in Tables 1 and 2 the two groups were well balanced regarding clinical characteristics and angiographic findings. There were no significant differences between groups. No differences between groups (8 controls, 9 PostC patients) were seen among patients with MaR in the upper quartile (data not shown).

**Table 1:** Patient characteristics in the control and PostC groups

**Variables**	**Control group**	**PostC group**	**p**
	**n = 35**	**n = 33**	
*At admission*			
Age, years (range)	62 (42–85)	63 (37–85)	0.96
Male sex	31 (89)	28 (85)	0.73
Body mass index, kg/m^2^ (IQR)	27 (25, 29)	27 (25, 31)	0.37
Ischemia time, minutes (IQR)	180 (141, 255)	165 (133, 202)	0.27
Current smokers	9 (26)	9 (27)	1.0
Hypertension	11(31)	5(15)	0.16
Previous angina	3 (9)	5 (15)	0.47
Diabetes mellitus*	10 (32)	9 (29)	1.0
*Treatment on admission*			
Aspirin,	3 (9)	1 (3)	0.61
Beta-blockers	4 (11)	1 (3)	0.36
ACE/ARB	6 (17)	2 (6)	0.26
Statins	3 (9)	2 (6)	1.0
*Treatment during angioplasty*			
Aspirin	35 (100)	31 (94)	0.23
Clopidogrel	34 (97)	31 (94)	0.61
Glycoprotein inhibitors	27 (77)	26 (79)	1.0
Opioids	26 (74)	28 (85)	0.37
*Treatment at discharge*			
Aspirin, n (%)	35 (100)	32 (97)	0.49
Clopidogrel, n (%)	35 (100)	33 (100)	1.0
Beta-blockers, n (%)	34 (97)	33 (100)	1.0
ACE/ARB, n (%)	20 (57)	21 (61)	0.64
Statins, n (%)	34 (97)	32 (97)	1.0

Data are presented as median and quartiles for continuous variables except age which is median and range, or number of patients and percentage for dichotomous variables. *ACE* Angiotensin converting enzyme inhibitor; *ARB* Angiotensin receptor blocker. * n = 31 both in Control and PostC.

**Table 2:** Angiographic data

**Variables**	**Control group**	**PostC group**	**p**
	**n = 35**	**n = 33**	
*Infarct related artery*			
LAD, n (%)	13 (37)	11 (33)	0.80
LCx, (%)	1 (3)	3 (9)	0.35
RCA, n (%)	21 (60)	19 (57)	1.0
Collateral flow grade 2 or 3, n (%)	6 (17)	4 (12)	0.74
*Number of vessels*			
one-vessel disease	22 (63)	21 (64)	1.0
two-vessel disease	11 (31)	10 (30)	1.0
three-vessel disease	2 (6)	2 (6)	1.0
Abnormally contracting segments (%)	25 (15, 34)	29 (17, 38)	0.43
Direct stenting, n (%)	2 (6)	0 (0)	0.49
Thrombectomy, n (%)	2 (6)	0 (0)	0.49
Bare metal stent, n (%)	35 (100)	32 (97)	0.49
TIMI flow grade 3 after PCI, n (%)	30 (86)	31 (94)	0.43

Data are presented as median and quartiles for continuous variables except age which is median and range, or number of patients and percentage for dichotomous variables. *LAD* Left anterior descending coronary artery; *RCA* Right coronary artery; *LCx* Left circumflex coronary artery; *TIMI* Thrombolysis in myocardial infarction; *PCI* Percutaneous coronary intervention.

### Infarct size and left ventricular ejection fraction

**Figure 2:**
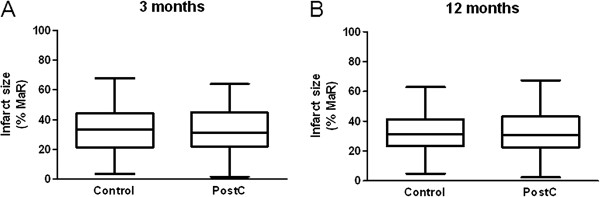
Box-plot of infarct size in relation to myocardium at risk (MaR) for the overall study population (n = 68) at (A) three and (B) 12 months in the control group and the postconditioning (PostC) group.

**Figure 3:**
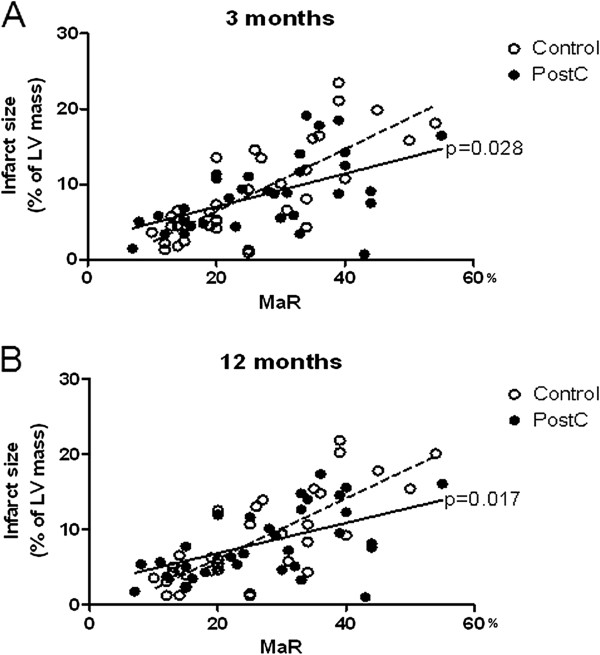
**Infarct size (expressed in relation to left ventricular mass) plotted against myocardium at risk (MaR) for the overall study population (n = 68) at (A) three and (B) 12 months in patients belonging to the control and postconditioning (PostC) groups.** Significant differences between the slopes of the regression lines of the two groups are indicated.

**Figure 4:**
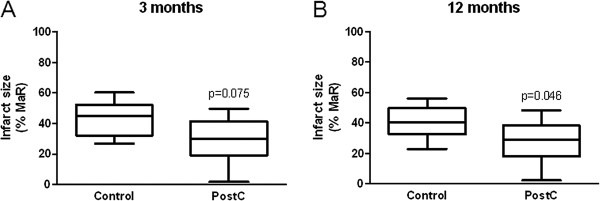
Box-plot of infarct size in patients within the upper quartile (n = 17) of myocardium at risk (MaR) at (A) 3 and (B) 12 months in the control group and the postconditioning (PostC) group.

Median infarct size, expressed as a percentage of MaR, at three and 12 months did not differ between the control and PostC groups (Figure 2). However, the slope of the regression lines for the final infarct size in relation to MaR differed significantly between the two groups, an observation that was consistent over time (Figure 3). Patients within the upper quartile of MaR (>35% of the LV) were therefore analyzed separately in line with the analysis performed after one week [8]. After three months PostC patients had a trend towards smaller infarct sizes than those in the control group a difference that became significant after 12 months (Figure 4). In contrast, there were no significant differences in infarct size in patients within the lower quartiles of MaR (data not shown). The intra- and interobserver variation between two blinded readers for infarct size measurement (n = 18) was 0.2 ± 1.0 and 0.1 ± 1.3 and for LV mass (n = 12) -0.8 ± 7.0 and −0.4 ± 6.8 (mean difference ± SD), respectively.

**Figure 5:**
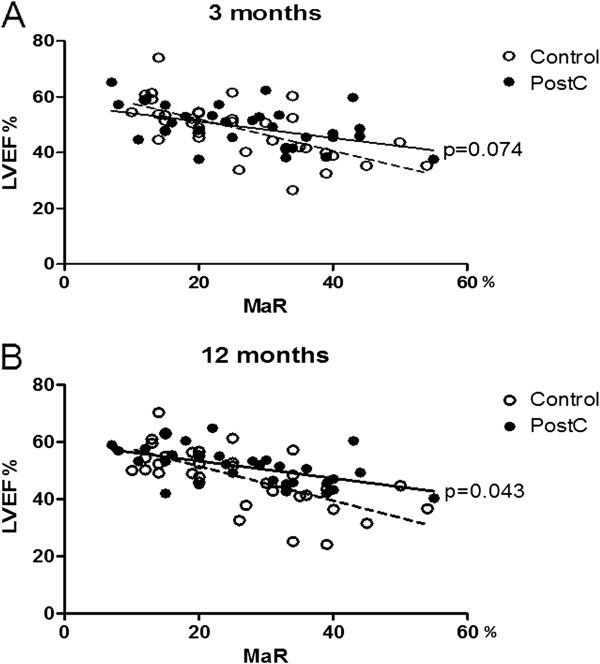
**Left ventricular ejection fraction (LVEF) plotted against myocardium at risk (MaR) for the overall study population (n = 68) at (A) three and (B) 12 months in patients belonging to the control and postconditioning (PostC) groups.** P-values between the slopes of the regression lines of the two groups are indicated.

Median LVEF for the whole study population did not differ between the control and PostC groups. The slope of the regression lines describing LVEF in relation to MaR had a trend towards improved LVEF after three months and differed significantly between the two groups after 12 months (Figure 5). In the group of patients in the upper quartile of MaR, LVEF was significantly higher in the PostC group than in the control group both after three and 12 months. LVEF at 12 months was 47% (43, 50) in the PostC group compared to 38% (34, 42; p < 0.01) in the control group.

### Remodeling data/parameters

LV mass index decreased significantly from one week to 12 months in both groups and there was no difference within the control and PostC groups regarding indexed volumetric CMR data at 3 and 12 months (Table 3). At one week PostC indexed volumes were slightly smaller compared to controls in the overall group. Infarct size, in absolute grams, decreased significantly between one week and 12 months in both groups. The mean decrease in absolute infarct size was 30% and 32% in the control and PostC group, respectively. The main decrease was seen between one week and 3 months. This also affected all other parameters that included infarct size in the calculation. Adverse LV remodeling occurred in only nine patients equally distributed between the two groups. End-diastole LV sphericity index for the entire study population did not differ between or within groups over time.

**Table 3:** CMR characteristics for Control and PostC, long-term follow-up

**Control, n=35**				**PostC, n=33**		
	**1 week**	**3 months**	**12 months**	**1 week**	**3 months**	**12 months**
**LV end-diastolic volume index, ml/m**^**2**^	89 (80, 99)	88 (77,103)	86 (70, 104)	79 (73, 84)	85 (77, 99)	79 (73, 91)
**LV end-systolic volume index, ml/m**^**2**^	49 (37, 56)	44 (35, 58)	39 (33, 57)	43 (36, 55)	43 (37, 55)	40 (32, 48)
**Sphericity index, end-diastole**	0.39 (0.35, 0.42)	0.39 (0.32, 0.47)	0.38 (0.33, 0.44)	0.38 (0.34, 0.45)	0.40 (0.35, 0.44)	0.37 (0.33, 0.44)
**LV-mass index, g/m**^**2**^	68 (60, 75)	61 (55, 69)**	60 (55, 67)***, ^#^	65 (59, 75)	59 (56, 70)***	57 (54, 68)***
**Infarct size, % of LV mass**	8.0 (5.5, 14.1)	6.6 (4.3, 13.6)***	6.0 (4.3, 13.1)***	9.9 (5.5, 14.9)	8.8 (5.0, 11.5)***	7.6 (4.9, 12.2)***
**Infarct size, g**	12.3 (7.5, 23.8)	9.1 (5.4, 21.1)***	7.8 (4.9, 19.8)***, ^##^	11.2 (7.7, 25.5)	10.1 (6.2, 18.8)***	10.6 (5.4, 16.9)***
**Infarct size, % of ACS**	39 (27, 54)	33 (21, 44)***	31 (23, 41)***	41 (26, 54)	31 (22, 45)***	31 (23, 43)***
**Microvascular obstruction (LGE), % of LV mass**	2.0 (1.4, 2.8)	-	-	1.8 (1.1, 2.8)	-	-

Median and inter quartile range. *ACS* Abnormally contracting segments; *LGE* Late gadolinium enhancement, *LV* Left ventricle. * p < 0.05; ** p < 0.01; *** p < 0.001 vs. 1 week; # p < 0.05, ## p < 0.01 vs. 3 months. Wilcoxon sign rank test with Bonferroni correction.

## Discussion

In this one year follow-up of patients with a first time STEMI, PostC did not reduce infarct size in relation to MaR or improve LVEF in the overall study population. On the other hand, there was a sustained beneficial impact in patients with large MaR as reflected by improved LVEF and smaller infarct size which was persistent during 12 months of follow up. The study therefore suggests that PostC exerts long-term beneficial effects among patients with large MaR.

The first observations of the possibility to reduce infarct size by means of PostC were reported by Staat et al. [9] and Laskey et al. [21]. They reported a reduced creatinine kinase levels and improved coronary flow reserve and ST-resolution, respectively. In 2012 two studies were published with conflicting results [11,22]. Frexia et al. were unable to verify any effect of PostC compared with controls while Thuny et al. confirmed their original findings of a favorable impact on infarct size and edema [9,14]. These discrepancies may have several explanations. The patients in the latter study were younger with less comorbidity and were only subjected to direct stenting but no thrombectomy. This is also emphasized in a recent meta-analysis [23]. Similar differences may also explain the results of the present study. Our population was older (median age of 62 years) and the proportion of patients with diabetes was higher than in the study by Thuny et al. but the potential impact of comorbidities has so far not been fully understood. Direct stenting and thrombectomy may be of importance by decreasing distal embolisation. Since direct stenting (n = 2) and thrombectomy (n = 2) was performed in only four of the control patients it should be of minor importance for the present results.

The first original report of the present study material, using CMR for determination of infarct size in relation to MaR one week after the acute myocardial infarction, PostC did not influence infarct size in patients with small risk areas while there was a significant reduction among those with MaR in the highest quartile [8]. In this long-term follow-up, it was found that infarct size and LVEF did not differ in PostC patients in the overall study group when re-examined after three and 12 months following the acute event. The slopes of the regression lines for infarct size and LVEF as a function of MaR did, however, differ at these times of observation indicating a sustained benefit for patients with large risk areas. Based on this observation, infarct size and LVEF was analysed in patients within the upper quartile of MaR, reflecting large threatening infarcts. Infarct size was still significantly smaller in the PostC group within this quartile. This could be expected taking previously reported data on infarction absorption and acute modulation of the reperfusion injury into account [12,24,25]. More interestingly, LVEF was higher after 12 months among patients in the PostC than in the control group. Our data support and extend the previous long-term observation by Thibault et al. [14] using echocardiography that LVEF was higher in the PostC group after one year. Our study is the first to demonstrate both LVEF and infarct size determined with CMR one year after the initial event.

The finding that the beneficial effect is found among patients with large MaR is in accordance with previous observations. The present study had a comparably small proportion of LAD infarctions and consequently a mean MaR of 27% in comparison with a mean MaR in the magnitude of 35-40% in previous reports demonstrating a beneficial effect of PostC [7,9,11,14]. Taken together, available data might indicate that PostC is effective in patients with large areas of ischemic myocardium and that the effect is maintained during at least one year following the acute event.

CMR is considered as the reference method for the determination of cardiac volumes and final infarct size due to its accuracy, reproducibility and ability to detect small morphological and functional changes [26,27]. LV remodeling after myocardial infarction is an important prognostic factor for progression to heart failure and subsequent mortality [28]. Multiple factors contribute to the remodeling process including infarct size, microvascular obstruction, patency of the infarct related artery and baseline LVEF [29,30]. Recent studies have used CMR as a tool for identifying predictors of remodeling in reperfused STEMI populations. Lund et al. [31] demonstrated that an infarct size ≥24% of the LV mass predicted remodeling with high sensitivity and specificity. Masci et al. concluded that infarct size rather than location and salvage index predicted LV remodeling in STEMI patients [32,33]. Only nine of the patients in the present study met the remodeling criteria (a consistent increase in ESV >15%) and small infarct sizes is the most reasonable explanation [24,31,32]. In addition the vast majority of the present patients were treated with beta-blockers and angiotensin converting enzyme inhibitors, pharmacological agents known to counteract remodeling. This made the group of patients with adverse remodeling too small for accurate analysis of possible predictors for that process. The end-diastolic sphericity index, which indicates LV cavity remodeling, did not change over time within groups indicating that there was no overall adverse long-term remodeling. The significant decrease in LV mass between the first and second CMR examination can be explained by resorption of edema in the infarct territory and replacement with fibrosis. The observation that absolute infarct size decreased in only the control group between three and 12 months is surprising considering existing data [24,25]. The difference was only 1.3 grams in median but there is no obvious pathological or physiological data supporting the notion that a necrotic mass may decrease after one year.

### Limitations

An important limitation of the present study is the small study population which may result in a type II statistical error regarding the detection of minor benefits of PostC. Although, the method of relating infarct size to MaR and the strong reproducibility of CMR reduces the number of patients needed to detect differences between groups. The number of patients was based on a power calculation assuming a 20% relative reduction of infarct size by PostC [8]. There was a 24% loss to follow up, which might induce the risk of selection bias. However, this risk is minimized due to the fact that the loss was evenly distributed with 11 patients in the PostC and 10 patients in the control group.

The lack of using direct stenting and presence of collaterals could potentially affect infarct size and thereby the result of the study. Importantly however, these parameters were equally distributed between the groups. Still the findings of persistent infarct size reduction and improved LVEF among patients within the upper quartile of MaR in the PostC group is of considerable and confirmatory interest. These findings should be considered when planning future studies.

## Conclusions

In this long-term follow-up study PostC did not reduce infarct size in relation to MaR or improved LVEF in the overall study population. However, the present data suggest that PostC exerts long-term beneficial effects in patients with large MaR thereby extending previously published short-term observations.

## Competing interests

The authors declare that they have no competing interests.

## Authors’ contributions

All authors participated in the design and coordination of the study, helped to draft the manuscript, read and approved the final manuscript. PS and JP also performed the statistical analysis.

## Pre-publication history

The pre-publication history for this paper can be accessed here:

http://www.biomedcentral.com/1471-2261/13/22/prepub

## References

[B1] Lloyd-Jones D Adams RJ Brown TM Carnethon M Dai S De Simone G Ferguson TB Ford E Furie K Gillespie C Go A Greenlund K Haase N Hailpern S Ho PM Howard V Kissela B Kittner S Lackland D Lisabeth L Marelli A McDermott MM Meigs J Mozaffarian D Mussolino M Nichol G Roger VL Rosamond W Sacco R Sorlie P Executive summary: heart disease and stroke statistics–2010 update: a report from the American Heart Association Circulation 2010 121 948 954 20177011 10.1161/CIRCULATIONAHA.109.192666

[B2] Shah SR Hochberg CP Pinto DS Gibson CM Reperfusion strategies for ST-elevation myocardial infarction Curr Cardiol Rep 2007 9 281 288 10.1007/BF02938376 17601394

[B3] Terkelsen CJ Christiansen EH Sorensen JT Kristensen SD Lassen JF Thuesen L Andersen HR Vach W Nielsen TT Primary PCI as the preferred reperfusion therapy in STEMI: it is a matter of time Heart 2009 95 362 369 19218262 10.1136/hrt.2007.139493

[B4] Keeley EC Boura JA Grines CL Primary angioplasty versus intravenous thrombolytic therapy for acute myocardial infarction: a quantitative review of 23 randomised trials Lancet 2003 361 13 20 10.1016/S0140-6736(03)12113-7 12517460

[B5] Heusch G Boengler K Schulz R Inhibition of mitochondrial permeability transition pore opening: the holy grail of cardioprotection Basic Res Cardiol 2010 105 151 154 10.1007/s00395-009-0080-9 20066536

[B6] Yellon DM Hausenloy DJ Myocardial reperfusion injury N Engl J Med 2007 357 1121 1135 10.1056/NEJMra071667 17855673

[B7] Lonborg J Kelbaek H Vejlstrup N Jorgensen E Helqvist S Saunamaki K Clemmensen P Holmvang L Treiman M Jensen JS Cardioprotective effects of ischemic postconditioning in patients treated with primary percutaneous coronary intervention, evaluated by magnetic resonance Circ Cardiovasc Interv 2010 3 34 41 10.1161/CIRCINTERVENTIONS.109.905521 20118154

[B8] Sorensson P Saleh N Bouvier F Bohm F Settergren M Caidahl K Tornvall P Arheden H Ryden L Pernow J Effect of postconditioning on infarct size in patients with ST elevation myocardial infarction Heart 2010 96 1710 1715 10.1136/hrt.2010.199430 20956486

[B9] Staat P Rioufol G Piot C Cottin Y Cung TT L’Huillier I Aupetit JF Bonnefoy E Finet G Andre-Fouet X Postconditioning the human heart Circulation 2005 112 2143 2148 10.1161/CIRCULATIONAHA.105.558122 16186417

[B10] Zhao ZQ Corvera JS Halkos ME Kerendi F Wang NP Guyton RA Vinten-Johansen J Inhibition of myocardial injury by ischemic postconditioning during reperfusion: comparison with ischemic preconditioning Am J Physiol Heart Circ Physiol 2003 285 H579 H588 12860564 10.1152/ajpheart.01064.2002

[B11] Thuny F Lairez O Roubille F Mewton N Rioufol G Sportouch C Sanchez I Bergerot C Thibault H Cung TT Post-conditioning reduces infarct size and edema in patients with ST-segment elevation myocardial infarction J Am Coll Cardiol 2012 59 2175 2181 10.1016/j.jacc.2012.03.026 22676937

[B12] Vinten-Johansen J Shi W Perconditioning and postconditioning: current knowledge, knowledge gaps, barriers to adoption, and future directions J Cardiovasc Pharmacol Ther 2011 16 260 266 10.1177/1074248411415270 21821526

[B13] Lacerda L Somers S Opie LH Lecour S Ischaemic postconditioning protects against reperfusion injury via the SAFE pathway Cardiovasc Res 2009 84 201 208 10.1093/cvr/cvp274 19666677

[B14] Thibault H Piot C Staat P Bontemps L Sportouch C Rioufol G Cung TT Bonnefoy E Angoulvant D Aupetit JF Long-term benefit of postconditioning Circulation 2008 117 1037 1044 10.1161/CIRCULATIONAHA.107.729780 18268150

[B15] Feild BJ Russell RO Jr Dowling JT Rackley CE Regional left ventricular performance in the year following myocardial infarction Circulation 1972 46 679 689 10.1161/01.CIR.46.4.679 5072769

[B16] Rentrop KP Cohen M Blanke H Phillips RA Changes in collateral channel filling immediately after controlled coronary artery occlusion by an angioplasty balloon in human subjects J Am Coll Cardiol 1985 5 587 592 10.1016/S0735-1097(85)80380-6 3156171

[B17] World Medical Association declaration of Helsinki Recommendations guiding physicians in biomedical research involving human subjects JAMA 1997 277 925 926 9062334

[B18] Heiberg E Sjogren J Ugander M Carlsson M Engblom H Arheden H Design and validation of segment–freely available software for cardiovascular image analysis BMC Med Imaging 2010 10 1 13 10.1186/1471-2342-10-1 20064248 PMC2822815

[B19] Heiberg E Ugander M Engblom H Gotberg M Olivecrona GK Erlinge D Arheden H Automated quantification of myocardial infarction from MR images by accounting for partial volume effects: animal, phantom, and human study Radiology 2008 246 581 588 18055873 10.1148/radiol.2461062164

[B20] Kono T Sabbah HN Rosman H Alam M Jafri S Goldstein S Left ventricular shape is the primary determinant of functional mitral regurgitation in heart failure J Am Coll Cardiol 1992 20 1594 1598 10.1016/0735-1097(92)90455-V 1452934

[B21] Laskey WK Yoon S Calzada N Ricciardi MJ Concordant improvements in coronary flow reserve and ST-segment resolution during percutaneous coronary intervention for acute myocardial infarction: a benefit of postconditioning Catheter Cardiovasc Interv 2008 72 212 220 10.1002/ccd.21583 18546233

[B22] Freixa X Bellera N Ortiz-Perez JT Jimenez M Pare C Bosch X De Caralt TM Betriu A Masotti M Ischaemic postconditioning revisited: lack of effects on infarct size following primary percutaneous coronary intervention Eur Heart J 2012 33 103 112 10.1093/eurheartj/ehr297 21846677

[B23] Zhou C Yao Y Zheng Z Gong J Wang W Hu S Li L Stenting technique, gender, and age are associated with cardioprotection by ischaemic postconditioning in primary coronary intervention: a systematic review of 10 randomized trials Eur Heart J 2012 33 3070 3077 10.1093/eurheartj/ehs265 22927556

[B24] Ganame J Messalli G Masci PG Dymarkowski S Abbasi K Van de Werf F Janssens S Bogaert J Time course of infarct healing and left ventricular remodelling in patients with reperfused ST segment elevation myocardial infarction using comprehensive magnetic resonance imaging Eur Radiol 2010 21 693 701 20865262 10.1007/s00330-010-1963-8

[B25] Engblom H Hedstrom E Heiberg E Wagner GS Pahlm O Arheden H Rapid initial reduction of hyperenhanced myocardium after reperfused first myocardial infarction suggests recovery of the peri-infarction zone: one-year follow-up by MRI Circ Cardiovasc Imaging 2009 2 47 55 10.1161/CIRCIMAGING.108.802199 19808564

[B26] Carlsson M Arheden H Higgins CB Saeed M Magnetic resonance imaging as a potential gold standard for infarct quantification J Electrocardiol 2008 41 614 620 10.1016/j.jelectrocard.2008.06.010 18817927

[B27] Kim RJ Fieno DS Parrish TB Harris K Chen EL Simonetti O Bundy J Finn JP Klocke FJ Judd RM Relationship of MRI delayed contrast enhancement to irreversible injury, infarct age, and contractile function Circulation 1999 100 1992 2002 10.1161/01.CIR.100.19.1992 10556226

[B28] Pfeffer MA Braunwald E Ventricular remodeling after myocardial infarction. Experimental observations and clinical implications Circulation 1990 81 1161 1172 10.1161/01.CIR.81.4.1161 2138525

[B29] Bogaert J Kalantzi M Rademakers FE Dymarkowski S Janssens S Determinants and impact of microvascular obstruction in successfully reperfused ST-segment elevation myocardial infarction. Assessment by magnetic resonance imaging Eur Radiol 2007 17 2572 2580 10.1007/s00330-007-0627-9 17361420

[B30] Geltman EM Ehsani AA Campbell MK Schechtman K Roberts R Sobel BE The influence of location and extent of myocardial infarction on long-term ventricular dysrhythmia and mortality Circulation 1979 60 805 814 10.1161/01.CIR.60.4.805 476885

[B31] Lund GK Stork A Muellerleile K Barmeyer AA Bansmann MP Knefel M Schlichting U Muller M Verde PE Adam G Prediction of left ventricular remodeling and analysis of infarct resorption in patients with reperfused myocardial infarcts by using contrast-enhanced MR imaging Radiology 2007 245 95 102 10.1148/radiol.2451061219 17885184

[B32] Masci PG Ganame J Francone M Desmet W Lorenzoni V Iacucci I Barison A Carbone I Lombardi M Agati L Relationship between location and size of myocardial infarction and their reciprocal influences on post-infarction left ventricular remodelling Eur Heart J 2011 32 1640 1648 10.1093/eurheartj/ehr064 21398642

[B33] Masci PG Ganame J Strata E Desmet W Aquaro GD Dymarkowski S Valenti V Janssens S Lombardi M Van de Werf F Myocardial salvage by CMR correlates with LV remodeling and early ST-segment resolution in acute myocardial infarction JACC Cardiovasc Imaging 2010 3 45 51 10.1016/j.jcmg.2009.06.016 20129530

